# A biofoundry workflow for the identification of genetic determinants of microbial growth inhibition

**DOI:** 10.1093/synbio/ysab004

**Published:** 2021-01-28

**Authors:** Alaster D Moffat, Adam Elliston, Nicola J Patron, Andrew W Truman, Jose A Carrasco Lopez

**Affiliations:** 1 Department of Molecular Microbiology, John Innes Centre, Norwich Research Park, Norwich, UK; 2 Department of Engineering Biology, Earlham Institute, Norwich Research Park, Norwich, UK

**Keywords:** biofoundry, automated testing workflow, *Pseudomonas*, biocontrol, *Streptomyces scabies*

## Abstract

Biofoundries integrate high-throughput software and hardware platforms with synthetic biology approaches to enable the design, execution and analyses of large-scale experiments. The unique and powerful combination of laboratory infrastructure and expertise in molecular biology and automation programming, provide flexible resources for a wide range of workflows and research areas. Here, we demonstrate the applicability of biofoundries to molecular microbiology, describing the development and application of automated workflows to identify the genetic basis of growth inhibition of the plant pathogen *Streptomyces scabies* by a *Pseudomonas* strain isolated from a potato field*.* Combining transposon mutagenesis with automated high-throughput antagonistic assays, the workflow accelerated the screening of 2880 mutants to correlate growth inhibition with a biosynthetic gene cluster within 2 weeks.

## 1. Introduction

Biofoundries are integrated automation platforms for the design, construction and characterization of organisms for biotechnology applications and research. They combine experimental approaches, such as mathematical and statistical modeling (i.e. design of experiments, DoE), computer-aided design (CAD) and analytical software, together with automated experimental platforms to enable high-throughput automated workflows. Over the last decade, many research institutions have established biofoundries to accelerate both fundamental and applied research (1). Previous to this, the use of automation in research labs was sporadic with uptake mainly limited to service laboratories performing repetitive workflows, such as diagnostics or genotyping.

The era of synthetic biology has applied engineering principles, such as standardization and predictive models to inform experimental design (2). This has enabled experiments to be scaled, making automated workflows not only attractive but necessary. Additional benefits of automation include improvements in the accuracy and reproducibility of experimentation (1, 3). A further tenet of engineering prescribed by synthetic biology is the progression of design-build-test-learn (DBTL) cycles, around which biofoundry workflows are typically centered. In the ‘design’ phase of the cycles, CAD tools are often used to assemble complex experimental designs, e.g. Ref. (4–6) and statistical techniques, such as DoE, are used to identify critical influencing factors and define the scale of the design space (7). Following this, automated workflows are used to assemble or ‘build’ the designs, typically from DNA. In the ‘test’ phase, specific analytic measurements are taken using techniques relevant to the phenotype or characteristic of interest. Finally, data are analyzed in the ‘learn’ phase and conclusions are used to inform the design phase of the subsequent cycle (1).

While the DBTL has clear applications in metabolic engineering (8), the applicability of biofoundries to the wider bioscience research community is not always obvious. The significant costs of establishing and maintaining biofoundries require an active user base. Barriers identified for offsetting the cost of newly established biofoundries include a limited awareness of the capabilities among potential users (1). The basic software and hardware infrastructure of biofoundries, combined with the associated expertise in large-scale experimental design can, however, be applied to a wide range of molecular and microbial workflows enabling biofoundries to become central research infrastructures.

Here, we demonstrate the broader applicability of biofoundries to established molecular and microbial methods through the development and application of automated workflows for the rapid and scalable creation and phenotypic screening of mutant libraries. The ability to identify single-gene determinants of inhibitory activity in random mutagenesis studies is limited only by library size, which presents a significant limitation in manual screening efforts. In this example, we describe workflows for the identification of genes associated with the inhibition of microbial growth. This leads to the identification of a biosynthetic gene cluster (BGC) in the genome of *Pseudomonas* sp. Ps652, recovered from a commercial potato field in Norfolk (UK). This isolate had previously been shown to display strong *in vitro* activity against *Streptomyces scabies* 87-22, a commercially relevant potato pathogen (9, 10). The determinants of *Pseudomonas* sp. Ps652 inhibition of *S. scabies* 87-22 were not evident from the Ps652 genome sequence or rational gene deletions and, given the large zones of inhibition observed on plates, it was not possible to use a standard 96 solid pin replicator to assay transposon mutants on the same plate alongside the pathogen. To overcome this constraint, we developed biofoundry workflows to generate and assay a library of random transposon mutants. This enabled the identification of a BGC associated with the production of a compound able to inhibit the growth of *S. scabies* 87-22. There is substantial interest in the role of microbial antagonism in the health of humans (11), animals (12) and plants (13), but it can be difficult to elucidate the genetic determinants of this antagonism, which can be complex and manifold. The workflow described here provides a template for the automated identification of these genes.

## 2. Materials and methods

### 2.1 Bacterial strains and media

*Pseudomonas* sp. Ps652ΔHCN, a cyanide null mutant of an environmental isolate from a commercial potato field in Norfolk, UK (GenBank Accession GCA_902497775.1) was used as the parent strain for transposon mutagenesis. *Pseudomonas* sp. Ps652ΔHCN was stored at −80°C in 925 µl L medium with 75 µl DMSO. *Streptomyces coelicolor* M145 and *S. scabies* 87-22 were stored as spore stocks that were grown to sporulation on potato dextrose agar, harvested in 20% glycerol and stored at −80°C. The same spore stock was used for the entire workflow.

All media used in this study were adjusted to pH 7.2. Rye Sucrose Agar (RSA): 60 g/l rye grains, 20 g/l sucrose, 20 g/l Agar was prepared by soaking rye grains in a 1/40 dilution of 10% chlorite solution for 4 min before rinsing with reverse osmosis purified water and germinating overnight. Germinated rye grains were then ground at high speed for 2 min with a hand blender, before being transferred to a water bath for 3 h at 50°C in Milli-Q water. This was then filtered through a sieve and muslin cloth before addition of sucrose and agar and sterilization by autoclaving. Malt Extract-Yeast Extract Maltose Medium (MYM): 10 g/l malt extract, 4 g/l yeast extract, 4 g/l maltose, 18 g/l bacteriological agar (Sigma-Aldrich, UK), made up to 1 l with tap water. Lennox Broth and Lennox Agar (L): 10 g/l tryptone, 5 g/l yeast extract, 5 g/l NaCl, 1 g/l D-glucose, 10 g/l Formedium agar (Lennox Agar), made up to 1 l with Milli-Q water. Lysogeny Broth (LB): 10 g/l tryptone, 5 g/l yeast extract, 10 g/l NaCl, made up to 1 l with Milli-Q water. Potato Dextrose Agar: 41 g/l potato dextrose agar (Formedium PDA0102S), made up to 1 l with Milli-Q water.

### 2.2 Production and selection of a *Pseudomonas* mutant library

*Pseudomonas* sp. Ps652ΔHCN was transformed by electroporation in 300 mM sucrose with the mariner transposon-containing plasmid pALMAR3 (14) and 100 µl was plated directly in single-well Nunc^TM^ OmniTray^TM^ plates (242811, Thermo Scientific, UK) containing L agar + 25 µg/ml tetracycline with a plating density that enabled automated colony picking. Plates were incubated overnight at 28°C for 16 h and resulting colonies were selected using the Hamilton Microlab STARplus (Hamilton, Bonaduz AG, Switzerland) equipped with one 96-channel pipetting head and eight individual single channels capable of liquid level detection, a light table and camera for colony picking, and grippers for plate movement steps (Supplementary Data S1). Methods were programmed using the Venus Three software (version 4.4.07740, Hamilton). Details of scripts, deck layout and associated Hamilton libraries are provided in Supplementary Data S2.

The detection and selection of the maximum number of viable colonies were enabled by a modified picking method using the EasypickII software (Version 1.0.2, Hamilton). A maximum number of 500 colonies from each plate were selected to generate the library. Criteria for colony picking were established to avoid cross-contamination and aberrant phenotypes. Colonies were defined with a minimal circularity factor of 0.05 and an area of 0.4–15 mm^2^. The liquid transfer and aspiration process were initially hampered by the presence of air bubbles formed by successive pipetting that hampered the autosensing of liquid levels, leading to inaccurate transfers. To overcome this, the pipette position was altered during aspiration and ejection of media. Suitable colonies were transferred using disposable 300 µl tips into liquid media (200 µl L + 25 µg/ml tetracycline) in 96-well microplates (4ti-0117, Brooks Life Science, UK) (Supplementary Data S2). Cultures were grown at 28°C, 250 rpm for 16 h. 15 µl DMSO was added to each well manually using a multichannel pipette, and plates were stored at −80°C until required.

### 2.3 Automated plating of *S. coelicolor* M145

Spores of *S. coelicolor* M145 were resuspended into Milli-Q water at a concentration of 35 μl spore stock per 80 ml H_2_O. A Hamilton Microlab STARplus, as described above, was used to dispense 20 µl of spore suspension into the center of each well of 120 24-well plates (Greiner Bio-One, 662102) containing 1 ml RSA per well. Spores were distributed using the on-deck shaker at 1500 rpm for 5 s. The plates were dried for 1 h before use in automated bioassays. Supplementary Data S3 provides the link to the automated spore plating method scripts, deck layout and associated Hamilton libraries.

### 2.4 Automated bioassays against *S. coelicolor* M145

The library of *Pseudomonas* sp. Ps652ΔHCN mutants were spiked onto the *S. coelicolor* M145 plates using a Hamilton Microlab STARplus as described above. Pipette tips were used to transfer droplets by submersion without aspiration followed by a brief touch of the agar surface using a liquid level setting of 0 mm. Plates were incubated at 28°C for 5 days before photographic imaging (Canon Ixus 175, Canon, Tokyo, Japan). Supplementary Data S4 provides the link to the bioassay method scripts, deck layout and associated Hamilton libraries.

### 2.5 Growth curves

Mutants of interest were streaked by hand to single colonies and grown overnight in 10 ml LB. Growth curves were performed by manually inoculating 5 µl of overnight culture diluted to an optical density at 600 nm (OD600) = 0.01 into 150 µl of LB medium in 96-well plates. Plates were incubated at 30°C with shaking at 200 rpm for 48 h with absorbance readings taken every 30 min. Data were acquired on a SPECTROstar Nano UV/Vis microplate reader (BMG Labtech, Ortenberg, Germany), and processed using Microsoft Excel and R (15). Three replicates were used per mutant.

### 2.6 Cross-streak assays

Cross-streak assays against *S. scabies* 87-22 were performed manually on MYM tap agar. *Streptomyces* spores were streaked in two parallel lines on the plate using a sterile toothpick, which were then ‘cross streaked’ at a 90° angle with overnight cultures of *Pseudomonas* sp. Ps652ΔHCN mutants of interest. All mutants were assayed in triplicate. Plates were incubated for 5 days at 28°C before photographic imaging (Canon Ixus 175, Canon, Tokyo, Japan).

### 2.7 Insertion site sequencing

Sites of transposon insertion were determined by polymerase chain reaction (PCR) as described in Ref. (16). DNA flanking the insertion sites was enriched in two rounds of amplification using primers specific to the ends of the mariner transposon element (Arb1b 5'-GGCCAGCGAGCTAACGAGACNNNNGATAT-3' and Arb-PCR 5′-CGCAAACCAACCCTTGGCAG-3′) with an initial denaturation at 95°C for 3 min followed by 30 cycles of: 95°C for 15 s, 38°C for 30 s and 72°C for 90 s, followed by a final extension of 72°C for 3 min. Products were purified, eluted in 30 µl sterile water and used as template for a second round of PCR with degenerate primers that anneal to chromosomal sequences ﬂanking the transposon (Arb1 5′-GGCCAGCGAGCTAACGAGAC-3′ and Almar3-seq 5′-ACATATCCATCGCGTCCGCC-3′) with an initial denaturation of 95°C for 3 min followed by 30 cycles of 95°C for 30 s, 56°C for 30 s, and 72°C for 90 s, followed by a final extension of 72°C for 3 min. Amplicons were purified using a QIAquick Spin PCR Purification Kit (Qiagen, Hilden, Germany) as per the manufacturer’s instructions and sequenced by Sanger sequencing (Eurofins Genomics, Luxembourg City, Luxembourg) using the Almar3-seq primer (5′-ACATATCCATCGCGTCCGCC-3′).

## 3. Results

### 3.1 Automated production of a *Pseudomonas* sp. Ps652ΔHCN mutant library

Prior analysis of the *Pseudomonas* sp. Ps652 genome determined that it features no BGCs for known antimicrobial compounds (10), with the exception of a hydrogen cyanide (HCN) BGC (17). However, in preliminary experiments (unpublished data), we determined that HCN production in *Pseudomonas* sp. Ps652 only partly explained inhibition of *S. scabies* growth and that a non-volatile compound was contributing to inhibition. Therefore, by using a cyanide null mutant, Ps652ΔHCN, as the parent strain for the generation of a transposon mutant library, we predicted the screen would identify new genes relevant to the inhibitory phenotype.

**Figure 1.**
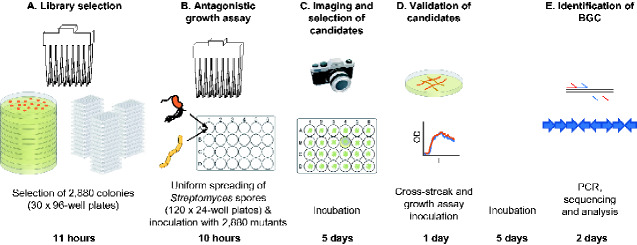
A Biofoundry workflow for the automated screening of microbial interactions. A mutant library of *Pseudomonas* sp. Ps652ΔHCN was created by automated selection of 2880 colonies (**A**). These were used in an automated bioassay with *S. coelicolor* M145 (**B**) and image analyzed after 5 days of incubation (**C**). Candidate strains were validated in growth assays and against the target pathogen, *S. scabies*, in a licensed laboratory (**D**) before the insertion site was identified by PCR and sequencing (**E**).

The primary bottleneck in the production of microbial mutant libraries is the selection of colonies and their subsequent curation. Manual colony-picking and subsequent screening of the resultant library is a tedious and time-consuming task with a consequential risk of human error and repetitive strain injury. We, therefore, automated the selection, culture and screening of a library of *Pseudomonas* sp. Ps652ΔHCN mutants to which the mariner transposon-containing plasmid pALMAR3 (14) had been delivered. Mariner type-transposons consist of a transposase gene flanked by inverted tandem repeats that insert their DNA cargo into the target genome at TA sites (18); the Ps652 genome contains 228 924 TA sites meaning that insertions of the transposase cargo DNA occur approximately randomly across the genome*.* The use of automation enabled the identification and selection of 2880 colonies (30 × 96-well plates) in 11 h with the added benefits of colony traceability (a digital record that links the original position of colonies to plate position for verification) and walk-away time (Figure 1A, Supplementary Data S2). The workflow was optimized to avoid colonies with aberrant morphologies and to prevent cross-contamination from neighboring colonies, facilitating scalability. The selection of 2880 mutants was based on the number of accessory coding sequences typically found in environmental *Pseudomonas* strains (19), however, the experiment is easily scalable.

### 3.2 Automated identification of *Pseudomonas* sp. Ps652 mutants unable to inhibit growth of *S. coelicolor* M145

*Streptomyces coelicolor* M145 was used in automated bioassays as a non-pathogenic proxy for *S. scabies* 87-22, which we were unable to work with in the biofoundry due to plant health license limitations. *Streptomyces scabies* and *S. coelicolor* both belong to clade II of a genome-based phylogeny for the *Streptomyces* genus (20), and preliminary work indicated that both were inhibited by Ps652ΔHCN (data not shown). Given the large zones of inhibition observed in pilot experiments, it was not possible to assay the mutant library on plates containing pathogen inoculum using a standard 96-pin replicator. We, therefore, developed an automated assay to assess the ability of 2880 *Pseudomonas* sp. Ps652ΔHCN mutants to affect the growth of *S. coelicolor* M145 using 24-well plates. This automated workflow consists of two steps. Firstly, the uniform spread of *Streptomyces* spores to each well (Figure 1B;  Supplementary Data S3) and, secondly, inoculation with *Pseudomonas* sp. Ps652ΔHCN (Figure 1B;  Supplementary Data S4). The automated assay required the optimization of (i) pipetting settings to minimize the time taken for the transfer of spore solution and plate inoculation, (ii) inoculation position (spiking) settings to enable the use of different volumes of agar in 24-well plates, (iii) lid-gripping and plate-gripping positions for accurate and rapid transfer of plates to stacks, and (iv) the speed (rpm) and time (s) for which plates were shaken to achieve a reproducibly homogenous spread of spores in every individual well. We also noted that it was essential to allow the spore solution to dry on the plates for 60 min before spiking the mutant library, to prevent Ps652 mutants from colonizing the entire well.

**Figure 2.**
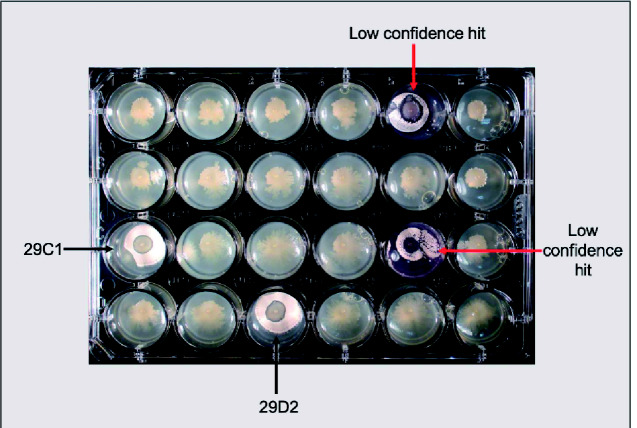
Representative image of a 24-well plate showing both low-confidence and high-confidence candidates, including 29C1 and 29D2, which were selected for further analysis.

The optimized bioassay was performed across 120 24-well plates and took ∼10 h to set up. Following incubation at 28°C for 5 days, the plates were imaged to identify mutants unable to inhibit *S. coelicolor* M145 growth (Figure 1C). Mutants of interest we defined as those in which *S. coelicolor* M145 grew immediately adjacent to the pseudomonad. High-confidence mutants were those in which the growth of the mutants themselves was uncompromised. Low-confidence mutants were defined as those in which *Pseudomonas* sp. Ps652ΔHCN growth appeared abnormal or in which a minimal zone of inhibition was visible (Figure 2). We identified 23 mutants of interest, which were further investigated in low-throughput screens.

### 3.3 Validation of candidates in cross-streak assays with *S. scabies*

**Figure 3.**
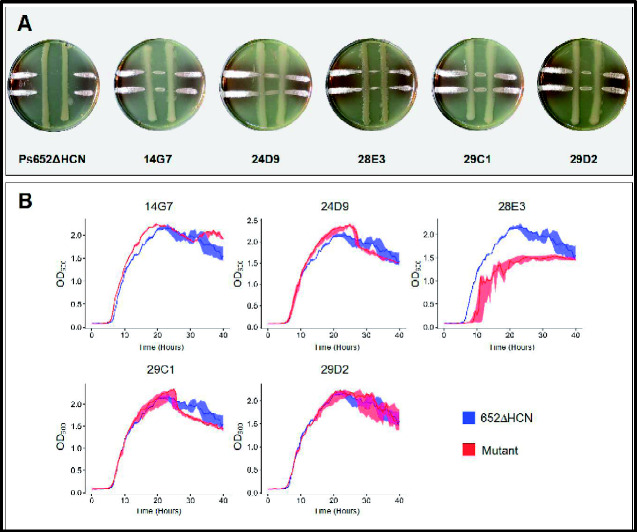
(**A**) Cross-streak assays of five *Pseudomonas* sp. Ps652ΔHCN mutants (vertical lines) against *S. scabies* 87-22 (horizontal lines). Images are representative of 3 replicates after incubation for five days. (**B**) Growth curves of *Pseudomonas* sp. Ps652ΔHCN (control) and five transposon mutants. Solid lines represent the median at each time point and the shaded area represent the maximum and minimum values; *n* = 3.

In order to validate the high-throughput screen, the 23 mutants identified in the high-throughput screen against *S. coelicolor* M145 were streaked to single colonies and used to inoculate cross-streak assays (see Section 2) with the target strain, *S. scabies* 87-22 (Figure 1D). Five mutant strains, 14G7, 24D9, 28E3, 29C1 and 29D2 were also unable to inhibit the growth of *S. scabies* 87-22 (Figure 3A). To rule out mutants that simply caused a Ps652 growth defect, growth curves were performed in liquid medium. Under these conditions, mutant 28E3 displayed a significant growth defect when compared with the parent strain and was excluded from further consideration (Figure 3B). Sequencing revealed that 28E3 had a transposon insertion in the *rfbA* gene, which encodes RmlA, a thymidylyltransferase involved in the biosynthesis of the cell wall component deoxythymidine diphosphate-L-rhamnose (21), highlighting the potential for off-target hits and false positives in this type of screen. All 22 remaining initial hits retained normal growth characteristics, including 14G7, 24D9, 29C1 and 29D2 (Figure 3B). Sites of transposon insertion for all 23 mutants are available in Supplementary Data S5.

### 3.4 Sequencing of transposon insertion sites identifies a candidate gene cluster

**Figure 4.**
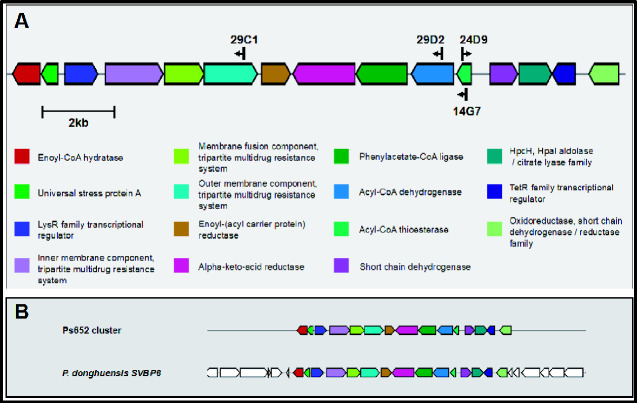
(**A**) Schematic of the *Pseudomonas* sp. Ps652 BGC indicating the location of transposon insertions (black arrows) and the conserved domains of the proteins encoded in this BGC. (**B**) Comparison of the cluster identified in *Pseudomonas* sp. Ps652 to a recently reported cluster linked to 7-hydroxytropolone production in *P. donghuensis* SVBP6.

The sites of transposon insertion were determined by PCR, and the four Ps652 mutants that were unable to inhibit the growth of both *S. coelicolor* M145 and *S. scabies* 87-22 all harbored transposon insertions within the same 8 kbp chromosomal region (Figure 4A). This region had not been identified as a BGC via antiSMASH 5.0 (22) or PRISM (23) analysis of the Ps652 genome, but did match a putative BGC that had been identified in *Pseudomonas donghuensis* SVBP6 in parallel with our experimental work. This was associated with antifungal activity and 7-hydroxytropolone biosynthesis in *P. donghuensis* (24). Comparative sequence analyses using MultiGeneBlast (25) indicated that the BGCs in *Pseudomonas* sp. Ps652 and *P. donghuensis* SVBP6 are highly similar, where they share the same genetic architecture (Figure 4B), as well as high identity/coverage scores for all genes (Supplementary Data S6).

## 4. Discussion

High-throughput screening is an emerging approach in many scientific fields and can be a useful tool in investigating microbial interactions (26–29). However, many researchers lack access to automation equipment or to programming expertise to enable existing equipment to be applied to their experimental workflow. As a result, high-throughput approaches often bypassed in favor of simple or low-throughput methodologies. This is particularly pertinent in microbial interactions, where the ability to scale bioassays holds immense promise but is impractical without the application of automation (27). In this study, we exemplified the relevance to biofoundries to the study of microbial interactions in the soil interactions, by conducting an automated screen that identified a BGC implicated in growth inhibition in just a few days of laboratory time. Such microbial interactions are of interest to agriculture, particularly the identification and characterization of plant-associated or rhizospheric soil bacteria capable of inhibiting the growth of specific plant pathogens (13, 30).

Advances in bioinformatic approaches to natural product discovery can be applied to such problems, aiding discovery of novel antimicrobial compounds in plant-associated bacteria (31). However, many existing bioinformatic approaches rely on homology to characteristic enzymes, such as polyketide synthases and non-ribosomal peptide synthetases, which represent roughly 65% of microbial metabolites used commercially (32). At present, it is likely that such approaches may be less reliable in identifying novel BGCs that display unusual and unprecedented genetic architectures, or that lack unifying features shared across the whole molecular class. Similarly, currently available bioinformatics tools can be of limited use in uncovering specialized metabolites where the biosynthesis is performed by a single protein, as has been shown previously for a volatile compound that is involved in biocontrol, 1-undecene (33). Consequently, novel compounds with societally relevant activities are still being discovered through bioactivity guided screening methods (34). As such, interesting phenotypes from select bacterial isolates still warrant investigation through screening against pathogens of interest.

To develop biofoundry workflows for studying microbial interactions, we focused on a *Pseudomonas* strain for which previous investigations had not identified BGCs of known antimicrobials (10, 24). The utility of our automated workflow was initially validated by growth curves and cross-streak assays (Figure 3), and subsequently by the identification of a BGC highly likely to produce the tropolonoid antimicrobial 7-hydroxytropolone. In parallel with our work, this BGC was identified through a transposon mutant screen of *P. donghuensis* SVBP6 (24) (Figure 4B). In that study, the authors associated the BGC with inhibitory activity of fungi and oomycetes, but not *Bacillus subtilis* or *Escherichia coli*. Our screen identified this BGC as being implicated in the inhibition of the filamentous Gram-positive bacterium *S. scabies* 87-22, a commercially important potato pathogen (35). Lacking the natural product class-defining proteins and being considerably different to published non-*Pseudomonas* tropolone BGCs (36), this cluster was not readily identified by genome mining tools.

The development of the workflow identified a number of important considerations and advantages of automated processes. The screening of bacterial libraries often relies on 96-solid pin replicators (37); however, some phenotypes are affected by the genotype or phenotype of the surrounding colonies on the plate. In the case of *Pseudomonas* sp. Ps652, mutant colonies that had lost the ability to produce a diffusible natural product were undetectable due to the production of the inhibitory natural product by neighboring colonies. Conducting the assays in individual wells of 24-well plates, an approach only practicable with automation, overcame this complication. Automating the assay also enabled the uniform distribution of *Streptomyces* spores, as well as the exact positioning and depth of the *Pseudomonas* inoculum. This used the liquid level detection feature and conductive tips to avoid the challenges of inconsistent agar depth. An additional advantage of automation is the ability to screen very large libraries. This is particularly important for single-gene determinants of phenotypes. Finally, the selection of false positives can be a potent obfuscating factor. These can increase due to inconsistent assay set-up as well as human errors or bias in visual screening. The ability to accurately and consistently inoculate assays and to program defined parameters for the automated identification of colony size and shape aided the robustness and reproducibility of the assay. Through the application of biofoundry workflows and approaches to experimentation, we were able to rapidly identify and prioritize a manageable number of high-confidence candidates for further characterization using manual approaches, ultimately implicating a BGC proposed to produce 7-hydroxytropolone in the biological control of the important potato pathogen *S. scabies* 87-22.

Most biofoundries are already engaged in the reconstruction and optimization of biosynthetic pathways to enable natural product biosynthesis. This study highlights their potential in the upstream experimental processes of gene discovery. In the workflow described here, the DBTL cycle consists of the selection of appropriate tools for the production of libraries, the construction and arraying of such libraries, phenotypic screens and genotyping of candidates, and, finally, data analysis to provide new knowledge of gene function. The outcomes may lead to either iterative cycles in which either library complexity or the robustness of the phenotypic screen are improved, or excitingly, link directly though the provision of novel genes to interconnected DBTL cycles in which the aim is biosynthesis. To aid this, critical comparisons of library production methods and the integration of high-throughput computer-aided quantitative phenotyping into biofoundry platforms would be highly beneficial.

## SUPPLEMENTARY DATA

Supplementary Data are available at *SYNBIO* Online.

## Supplementary Material

ysab004_Supplementary_Data
