# Supplementary material for: A biofoundry workflow for the identification of genetic determinants of microbial growth inhibition
Source: Synth Biol (Oxf). 2021 Jan 28;6(1):ysab004. doi: 10.1093/synbio/ysab004 (PMC7889406; doi:10.1093/synbio/ysab004)
Supplement: ysab004_Supplementary_Data [file ysab004_supplementary_data.docx]

**SUPPLEMENTARY DATA**

**A biofoundry workflow for the identification of biosynthetic gene clusters associated with microbial growth inhibition**

Alaster D. Moffat^1^, Adam Elliston^2^, Nicola J. Patron^2^, Andrew W. Truman^1*^, Jose A. Carrasco Lopez^2*^

^1^Department of Molecular Microbiology, John Innes Centre, Norwich Research Park, Norwich, NR4 7UH, UK

^2^Department of Engineering Biology, Earlham Institute, Norwich Research Park, Norwich, NR4 7UZ, UK

*Co-corresponding authors: jose.carrasco-lopez@earlham.ac.uk, andrew.truman@jic.ac.uk

**Supplementary Data 1. Deck layout of the Hamilton Microlab Star Plus**

The grippers (G) are used by the individual pipetting channels (P) to transport different plates to and from carriers (C) on the deck. The light table (Lt) was used to visualize colonies picked with conductive tips (Ct). Shakers (S) were used to spread *S. coelicolor* M145 spore solution onto 24-well plates.


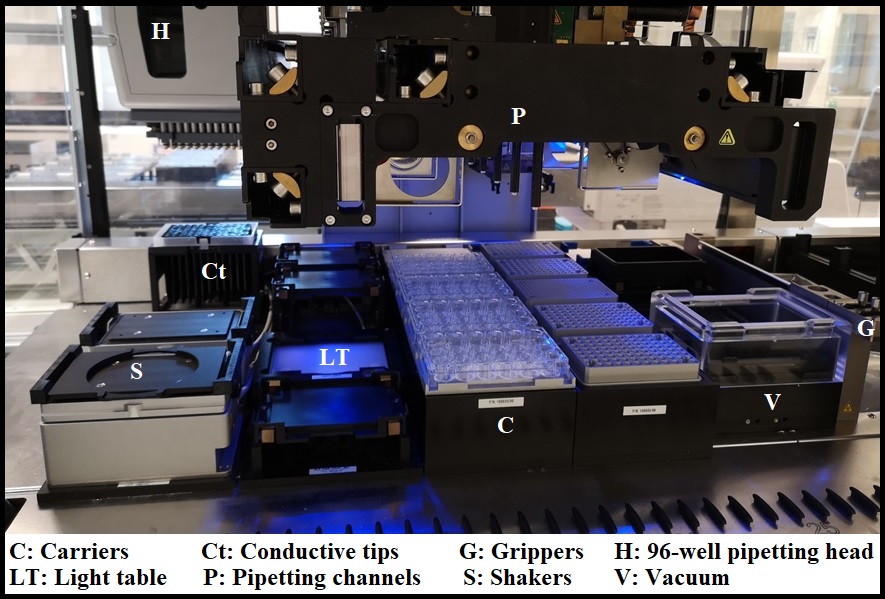


**Supplementary Data 2. Automated colony picking**

**A** Link to **Video 1** showing the automated colony picking of *Pseudomonas* sp. Ps652∆HCN mutants and arraying into 96-well plates: <https://youtu.be/cLQZDdMr0l0>

The scripts packages for the automated colony picking on the Hamilton Microlab STARplus are available on GitHub (<https://github.com/eibiofoundry/A-Biofoundry-workflow-to-identify-biosynthetic-gene-clusters.git>).

**B** Colonies meeting the selected criteria were identified using the Easypick software. Green-circled colonies are those meeting the criteria. Red-circled and orange-circled colonies are those that failed to meet the criteria and were not picked.


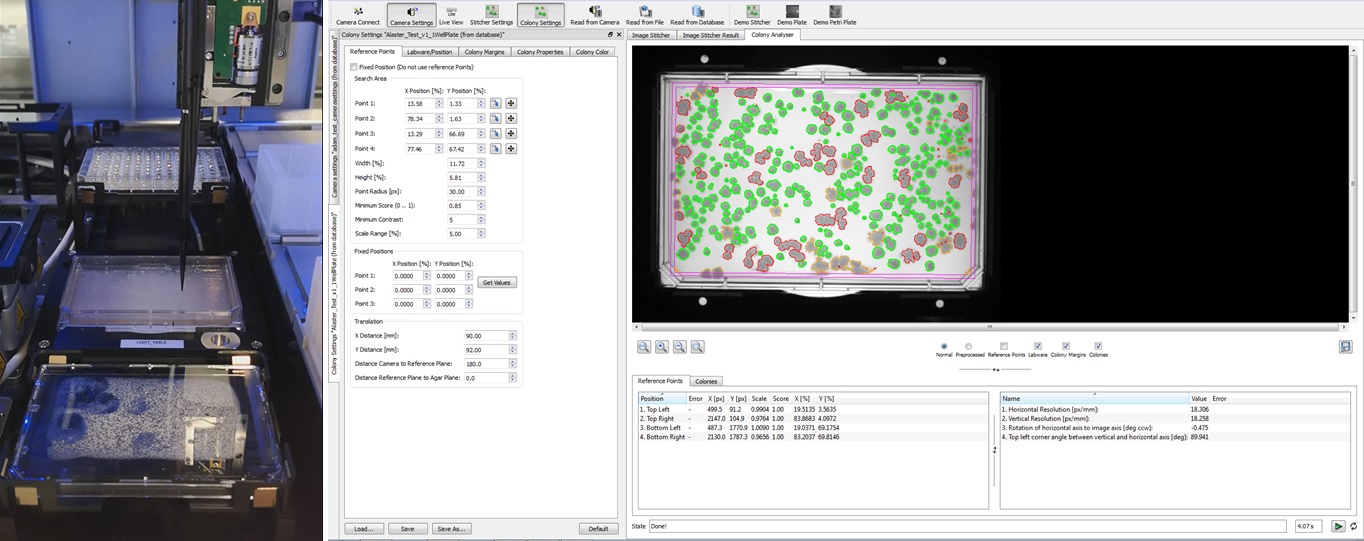


**Supplementary Data 3. Automated spore spreading**

Link to **Video 2** showing the automated distribution of *S. coelicolor* M145 spores on 24-well plates:

<https://youtu.be/3TEFXuciNu8>

The scripts packages for the automated spore spreading on the Hamilton Microlab STAR plus are available on GitHub (<https://github.com/eibiofoundry/A-Biofoundry-workflow-to-identify-biosynthetic-gene-clusters.git>).

**Supplementary Data 4. Automated inoculation**

Link to **Video 3** showing the automated inoculation of 24-well plates containing *S. coelicolor* M145 with the *Pseudomonas* sp. Ps652∆HCN mutant library: <https://youtu.be/nlIDy0Vf5ww>

The scripts packages for the automated inoculation of *Pseudomonas* sp. Ps652∆HCN mutant library on the Hamilton Microlab STAR plus are available on GitHub (<https://github.com/eibiofoundry/A-Biofoundry-workflow-to-identify-biosynthetic-gene-clusters.git>).

**Supplementary Data 5.** Table showing sites of transposon insertion in all 23 transposon mutants of Ps652∆HCN identified in the high-throughput screen against *S. coelicolor* M145.

| Mutant | Gene disrupted | Gene product annotation |
| --- | --- | --- |
| 4B3 | *ilvH* | Acetolactate synthase small subunit |
| 4A5 | *panB* | 3-methyl-2-oxobutanoate hydroxymethyltransferase |
| 10B3 | *leuB* | 3-isopropylmalate dehydrogenase |
| 14B12 | *argB* | Acetylglutamate kinase |
| 14G7 | *acdA1* | Acyl-CoA dehydrogenase |
| 15C7 | *trpA* | Tryptophan synthase alpha chain |
| 17A1 | *leuB* | 3-Isopropylmalate dehydrogenase |
| 17B4 | *ubiE* | Ubiquinone/menaquinone biosynthesis C- methyltransferase |
| 19C1 | *recA* | DNA recombination and repair protein |
| 20A6 | *bioC* | Malonyl-[acyl-carrier protein] O- methyltransferase |
| 20C5 | *hisF* | Imidazole glycerol phosphate synthase subunit |
| 21B6 | *mgtC/sapB* | MgtC/SapB family protein |
| 21E5 | *accB* | Biotin carboxyl carrier protein of acetyl-CoA carboxylase |
| 21F1 | *lcfB* | Long-chain-fatty-acid--CoA ligase |
| 21G8 | *trpA* | Tryptophan synthase alpha chain |
| 23D8 | *argB* | Acetylglutamate kinase |
| 24D3 | *metF* | 5,10-Methylenetetrahydrofolate reductase |
| 24D9 | *adc* | Acyl-CoA thioesterase |
| 25D12 | *trpB* | Tryptophan synthase beta chain |
| 26H7 | *dnaJ* | Hsp40 / Chaperone DnaJ |
| 28E3 | *rfbA* | Glucose-1-phosphate thymidylyltransferase |
| 29C1 | *oprM1* | Efflux transporter outer membrane subunit |
| 29D2 | *acdA1* | Acyl-CoA dehydrogenase |

**Supplementary Data 6.** Table showing similarity of proteins in the BSG clusters associated with 7-hydroxytropolone biosynthesis from *Pseudomonas* sp. Ps652 and *Pseudomonas* *donghuensis* SVBP6.

| *P. donghuensis* SVBP6 protein annotation | *P. donghuensis* SVBP6 Genbank Accession No. | Ps652 GenBank  Accession No. | Amino Acid Identity/Coverage |
| --- | --- | --- | --- |
| Enoyl-CoA hydratase | COO64_RS11460 | VVM48904.1 | 99/100 |
| Universal stress protein A | COO64_RS11465 | VVM48915.1 | 96/100 |
| LysR family transcriptional regulator | COO64_RS11470 | VVM48933.1 | 100/100 |
| Inner membrane component, tripartite multidrug resistance system | COO64_RS11475 | VVM48948.1 | 99/100 |
| Membrane fusion component, tripartite multidrug resistance system | COO64_RS11480 | VVM48972.1 | 96/100 |
| Outer membrane component, tripartite multidrug resistance system | COO64_RS11485 | VVM48995.1 | 94/100 |
| Enoyl-(acyl carrier protein) reductase | COO64_RS11490 | VVM49008.1 | 98/100 |
| Alpha-keto-acid reductase | COO64_RS11495 | VVM49029.1 | 98/100 |
| Phenylacetate-CoA ligase | COO64_RS11500 | VVM49057.1 | 98/96 |
| Acyl-CoA dehydrogenase | COO64_RS11505 | VVM49077.1 | 99/100 |
| Acyl-CoA thioesterase | COO64_RS11510 | VVM49090.1 | 99/100 |
| Short chain dehydrogenase | COO64_RS11515 | VVM49115.1 | 92/100 |
| HpcH, HpaI aldolase / citrate lyase family | COO64_RS11520 | VVM49135.1 | 96/100 |
| TetR family transcriptional regulator | COO64_RS11525 | VVM49157.1 | 97/100 |
| Oxidoreductase, short chain dehydrogenase / reductase family | COO64_RS11530 | VVM49178.1 | 93/100 |
